# Alteration of CXCR7 Expression Mediated by TLR4 Promotes Tumor Cell Proliferation and Migration in Human Colorectal Carcinoma

**DOI:** 10.1371/journal.pone.0027399

**Published:** 2011-12-13

**Authors:** Huanbai Xu, Qiong Wu, Shipeng Dang, Min Jin, Jingwei Xu, Yiji Cheng, Minglin Pan, Yugang Wu, Chunhui Zhang, Yanyun Zhang

**Affiliations:** 1 Shanghai Institute of Immunology, Institutes of Medical Sciences, Shanghai Jiao Tong University School of Medicine (SJTUSM) and Key Laboratory of Stem Cell Biology, Institute of Health Sciences, Shanghai Institutes for Biological Sciences, Chinese Academy of Sciences and SJTUSM, Shanghai, China; 2 Department of Endocrinology, The First Affiliated Hospital of Bengbu Medical College, Bengbu, China; 3 Department of Endocrinology, Second Affiliated Hospital of Nanjing Medical University, Nanjing, China; 4 Department of Surgery, The First People′s Hospital of Changzhou and The Third Affiliated Hospital of Soochow University, Changzhou, China; National Cancer Institute, United States of America

## Abstract

The link between inflammation and colorectal carcinoma has been acknowledged. However, the impact of bacterial lipopolysaccharide (LPS) binding to Toll-like receptor 4 (TLR4) on chemokine receptors in human colorectal carcinoma cells still remains to be elucidated. The present study shows that exposure to LPS elevated CXC chemokine receptor 7 (CXCR7) expression in colorectal carcinoma SW480 and Colo 205 cell lines expressing TLR4/myeloid differential protein (MD-2). CXCR7 is associated with SW480 cell proliferation and migration. However, exposure of SW480 and Colo 205 cells to LPS had no effect on CXCR4 expression. To further support the above results, the expression of TLR4, MD-2, and CXCR7 was analyzed in human colorectal carcinoma tissues. Higher rates of TLR4 (53%), MD-2 (70%), and CXCR7 (29%) expression were found in colorectal carcinoma tissues than in normal tissues. We demonstrated that the recombination of TLR4, MD-2 and CXCR7 strongly correlated with tumor size, lymph node metastasis and distant metastasis in colorectal carcinoma tissue samples (*p* = 0.037, *p* = 0.002, *p* = 0.042, resp.). Accordingly, simultaneous examination of the expression of TLR4, MD-2 and CXCR7 in cancer tissues of colorectal carcinoma may provide valuable prognostic diagnosis of carcinoma growth and metastasis. Interplay of TLR4, MD-2 and CXCR7 may be of interest in the context of novel immunomodulatory therapies for colorectal carcinoma.

## Introduction

Colorectal carcinoma is one of the most common cancers, which accounts for almost half a million deaths annually worldwide. Death of these patients results from uncontrolled metastatic disease, including liver, lymph nodes, or peritoneum metastases. Inflammation is considered a risk factor for many common malignancies including cancers of the lung [1], breast [2], and colorectum [3]. Chronic inflammatory bowel disease (IBD) such as chronic ulcerative colitis and Crohn's disease is associated with increased incidence of colorectal carcinoma as compared with the normal population [4]–[5]. The link between inflammation and colorectal carcinoma offers the possibility of identifying novel ways to prevent cancer. However, the molecular mechanisms whereby chronic inflammation predisposes to cancer remain elusive.

Different products of enterobacteriaceae and especially their lipopolysaccharides (LPS) may also contribute to perpetuation of the chronic colorectal inflammation [6]. Toll-like receptor 4 (TLR4), which is responsible for recognizing Gram-negative bacterial LPS, is upregulated and overexpressed in patients with IBD or colorectal carcinoma [7]–[8] whereas TLR4 normally is expressed at low level in the intestinal mucosa [1], [5], [9]. TLR4 is known to be implicated in the pathogenesis of chronic gastrointestinal disorders such as celiac disease [10] and IBD [11]. Additionally, TLR4 elicits appropriate immune activation in various types of cancer such as lung, ovary, stomach and colorectum. LPS binding induces the formation of a symmetric M-shaped TLR4-MD-2-LPS multimer composed of two copies of the complex to activate pro-inflammatory signaling pathways, which could induce expression of some chemoattractant receptors [12]–[13]. Nevertheless, little else is known of the involvement of TLR4 in the progression of colorectal carcinoma.

Initiation and progression of malignancies is the result of a series of complex processes that depend upon multiple and interactive factors [14]. Clinical and experimental studies indicate that CXC chemokines enhance immunity to tumor-associated antigens. However, they may also promote angiogenesis, proliferation and tumor cell invasion, such as the CXCL12 (SDF-1)/CXCR4 axis [15]. Recently, CXCR7 was identified as a second receptor for CXCL12. It was described to be present on the surface of many tumor cell types and on activated endothelial cells. Generally, but depending on cell type, CXCR7 was reported to be either a non-signaling receptor (decoy receptor) or a signaling receptor. However, the function and regulatory mechanisms of CXCR4/CXCR7 and the relationship between TLR4-MD-2 and CXCR4/CXCR7 in colorectal carcinoma is still unknown.

Our studies suggest that exposure to LPS elevates CXCR7 expression in a colorectal carcinoma SW480 and Colo 205 cell lines expressing TLR4/MD-2. Meanwhile, CXCR7/CXCL12 modulates tumor cell proliferation and migration. However, exposure of SW480 and Colo 205 cells to LPS had no effect on CXCR4 expression. Exposure of HT-29 cells with expression of only TLR4 to LPS had no effect on CXCR7 or CXCR4 expression. Furthermore, we found that combined expression of all three markers (TLR4, MD-2, and CXCR7) more strongly correlated with tumor size, lymph node metastasis and distant metastasis than did each of the three markers alone. Thus, recombination of TLR4, MD-2, and CXCR7 may prove to be valuable prognostic markers for predicting the proliferation and metastatic ability of colorectal carcinoma cells. An understanding of the relationships between microbial signals and colorectal carcinoma tissue might provide further clues for the development of new therapeutic strategies.

## Results

### Exposure of TLR4 to LPS elevates CXCR7 expression in colorectal carcinoma SW480 and Colo 205 cell lines

**Figure 1:**
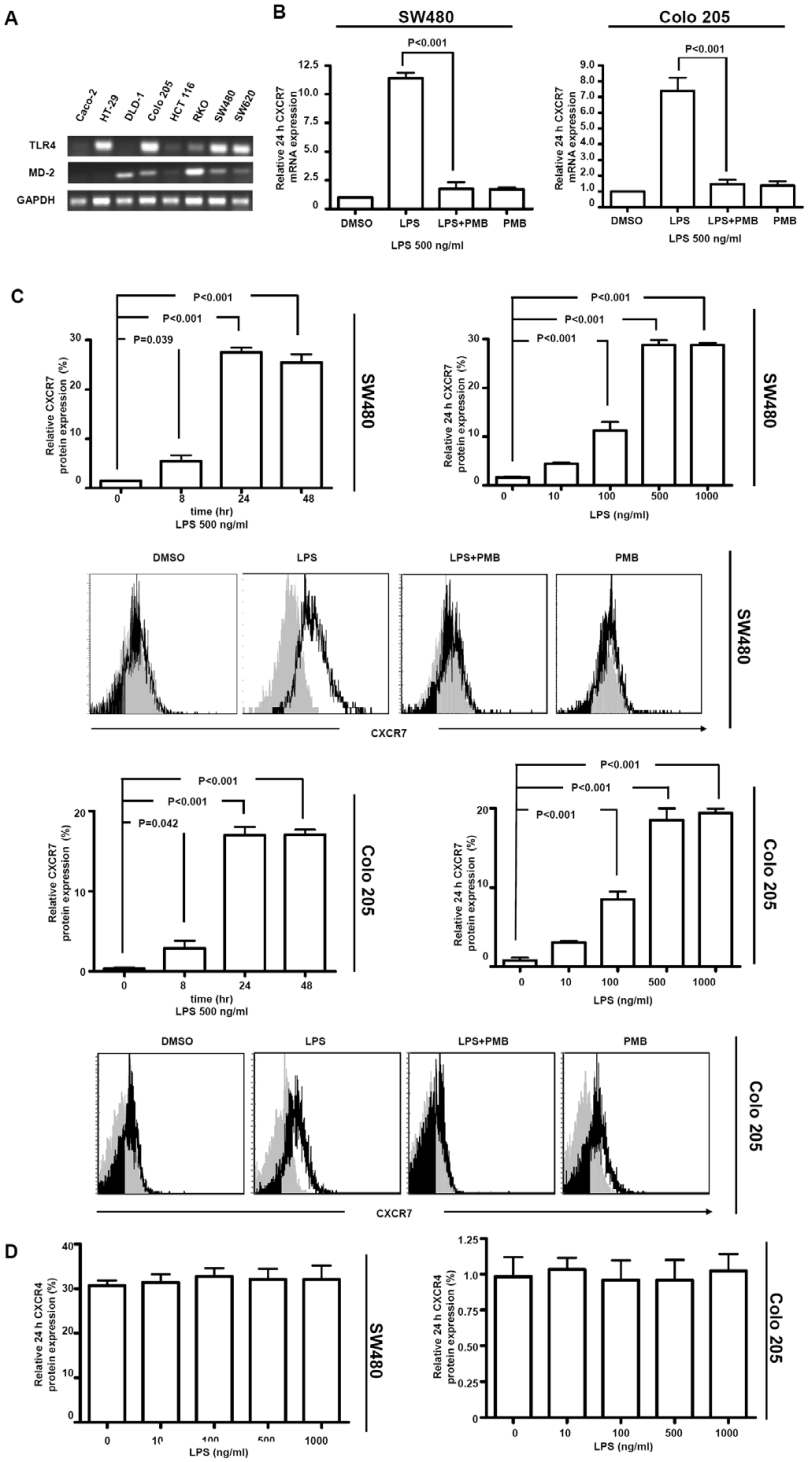
LPS-TLR4-MD-2 induced CXCR7 expression alteration in colorectal carcinoma cell line. A, Reverse transcriptase (RT)-PCR analysis (TLR4 and MD-2) on RNA isolated from 8 human colorectal carcinoma cell lines. B, LPS induced time- and dose-dependent CXCR7 and CXCR4 protein expression alterations. SW480 and Colo 205 cell lines were incubated with LPS (500 ng/ml) in the presence or absence of PMB (500 µg/ml), representative flow cytometric analysis of CXCR7 expression alterations were showed. C, LPS exposure induced a significant CXCR7 expression increase in total RNA conten. D, Exposure of SW480 and Colo 205 cell lines to LPS (500 ng/ml) had no effect on CXCR4 expression.

mRNA of colorectal carcinoma cell lines encoding TLR4 with its co-molecules, MD-2 were determined using RT-PCR. The analysis of RT-PCR products showed that TLR4 and MD-2 were constitutively expressed in 4 out of 8 human colorectal carcinoma cell lines, including Colo 205, RKO, SW480 and SW620, whereas TLR4 and MD-2 were present respectively in only two cell lines such as DLD-1 and HCT-29 (Fig. 1A). We tested two cell lines(SW480 and Colo 205) with expression of both TLR4 and MD-2 and one(HT-29) with expression of only TLR4.

CXCR7 normally was minimally expressed, whereas CXCR4 was expressed at high level in SW480 cells. Real-time quantitative-PCR and flow cytometry clearly indicated that the exposure of SW480 cells expressing TLR4/MD-2 to LPS induced CXCR7 expression alterations significantly (*p*<0.001, *p*<0.001, resp.; Fig. 1B, C). CXCR7 and CXCR4 were not expressed on Colo 205 cells. Similar results of LPS induced CXCR7 expression alterations significantly appeared in Colo 205 cells (*p*<0.001, *p*<0.001, resp. Fig. 1B, C).The natural peptide PMB is a well-known and potent antibiotic that binds and neutralizes bacterial endotoxin (LPS). In our study, PMB, the LPS inhibitor, inhibited the LPS-induced CXCR7 expression alterations (Fig. 1B, C). Maximal LPS effects were observed on CXCR7 protein expression after 24 h exposure to a dose of 500 ng/ml, representative examples of flow cytometry are shown (Fig. 1C). In contrast to LPS induced CXCR7 expression alterations, exposure of SW480 and Colo 205 cell lines to LPS had no effect on CXCR4 expression (Fig. 1D).

In addition, CXCR7 and CXCR4 were not expressed on HT-29 cells. Exposure of HT-29 cells with expression of only TLR4 to LPS had no effect on CXCR7 or CXCR4 expression (data not shown).

### Knockdown of MD-2 inhibits LPS-mediated CXCR7 expression

**Figure 2:**
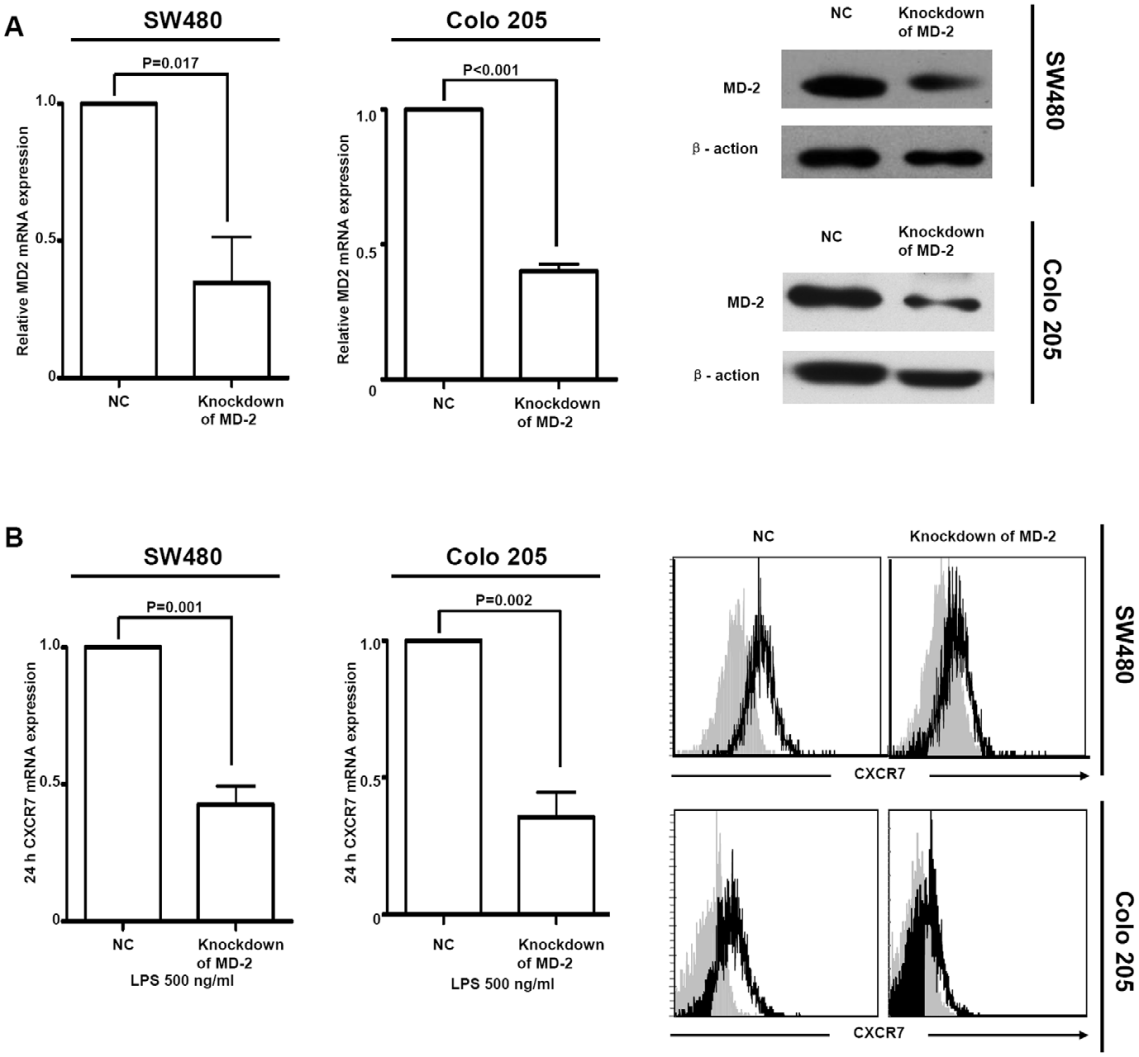
Knockdown effect of MD-2 on exposure of TLR4 to LPS in SW480 and Colo 205 cell lines. A, SW480 and Colo 205 cell lines were transfected transiently with siRNA or negative control sequence(NC). SW480 and Colo 205 cell lines transfected with the MD-2 siRNA sequence exhibited a marked reduction in MD-2 mRNA and protein level compared with NC. B, After LPS treatment, flow cytometry and real-time quantitative-PCR were performed. Knockdown of MD-2 inhibited LPS-mediated CXCR7 expression.

In order to investigate the role of MD-2 in LPS-mediated CXCR7 expression, we subjected SW480 and Colo 205 cell lines expressing TLR4/MD-2 to transient transfection with the siRNA specific for the MD-2 gene. 2 cell lines transfected with the MD-2 siRNA sequence exhibited a marked reduction in the abundance of the endogenous MD-2 mRNA and protein level compared with that in cells transfected with the negative control sequence (Fig. 2A).

Incubation of SW480 and Colo 205 cell lines transfected with the MD-2 siRNA sequence with LPS did not reveal an increase in CXCR7 mRNA and protein expression. In contrast, transfection with the negative control sequence in cells resulted in a marked increase in CXCR7 expression in response to LPS (*p* = 0.001, *p* = 0.002, resp.; Fig. 2B). These results suggest that MD-2 is essential for LPS-induced CXCR7 expression alteration.

### CXCR7 mediated by LPS modulates tumor cell proliferation and migration

Subsequently, we used a novel CXCR7-specific antagonist CCX771 and an endogenous CXCR7 ligand (CXCL12) to investigate the role of CXCR7 in regulating CXCL12-mediated colorectal carcinoma cell proliferation, apoptosis and migration.

**Figure 3:**
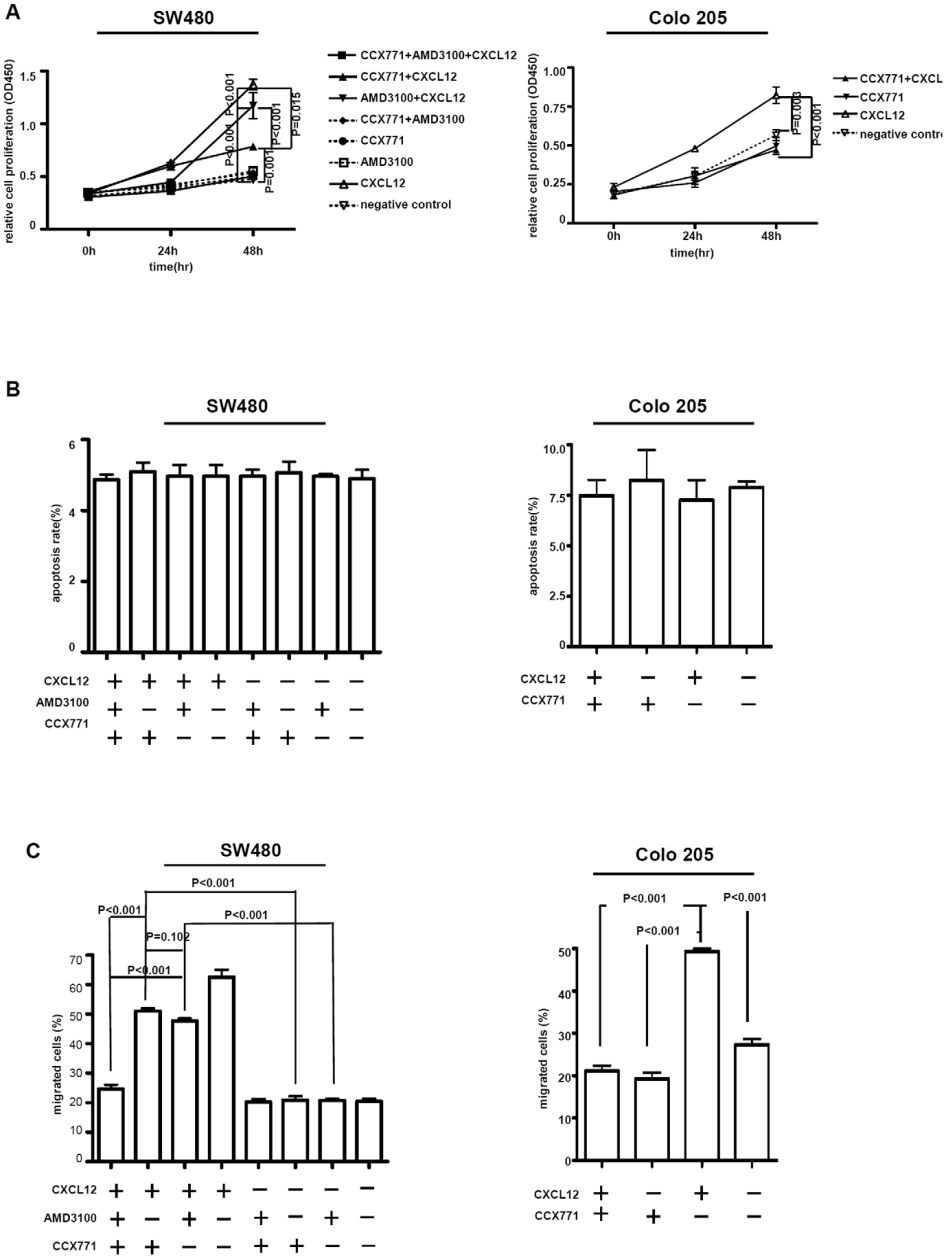
Proliferation, apoptosis and migration of CXCR7-positive SW480 and Colo 205 cell lines incubated with LPS. A, Incubation with LPS, SW480 cell line pretreated with AMD3100 (10 µg/ml) proliferated significantly in response to CXCL12 (100 ng/ml; 48 h), Colo 205 cell line also proliferated significantly in response to CXCL12. B, CXCL12 activation of its receptor CXCR7 did not exert an antiapoptotic effect. C, Pretreatment with AMD3100, SW480 cell line migrated significantly more in response to CXCL12 after 24 h of incubation with LPS, Colo 205 cell line also migrated significantly in response to CXCL12.

Incubation with LPS, the growth and viability of SW480 and Colo 205 cell lines for 24 h and 48 h in the presence or absence (control) of CXCL12 were examined. Because CXCR4 expressed on SW480 cells is another receptor for CXCL12, SW480 cells were pretreated (1 h) with a CXCR4 antagonist AMD3100 in some of the experiments. Upon CXCL12 stimulation, SW480 cells pretreated with AMD3100 proliferated significantly after 48 h (*p*<0.001, *p*<0.001, resp.; Fig. 3A). In addition, SW480 cells pretreated with CCX771 proliferated significantly after 48 h (*p* = 0.001, *p* = 0.015, resp.; Fig. 3A). However, the proliferative effect of CXCR7 is more than that of CXCR4 (*p*<0.001; Fig. 3A). At the same time, though CXCR4 was not expressed in Colo 205 cells incubated with LPS, CXCR7 significantly induced proliferation in Colo 205 cells ( *p* = 0.003; Fig. 3A). The proliferative effect of CXCR7 was blocked by a CXCR7 antagonist CCX771 (*p*<0.001; Fig. 3A).

No difference in apoptosis was detected in SW480 and Colo 205 cell lines grown with or without CXCL12 for up to 48 h, as demonstrated by the annexin V and propidium iodide binding assays (Fig. 3B). Therefore, CXCR7 expressed on colorectal carcinoma cells results in cell proliferation, but may not exert an antiapoptotic effect.

We also performed transwell migration assays to determine whether CXCR7 induces migration of colorectal carcinoma cells. After 24 h of incubation with LPS, not only CXCR7 but also CXCR4 expressed in SW480 cells significantly induced migration in the presence of CXCL12 (*p*<0.001, *p*<0.001, resp.; Fig. 3C). However, there was no significant difference in migration effect of CXCR7 and CXCR4 (*p* = 0.102; Fig. 3C). Incubated with LPS, CXCR7 significantly induced migration in Colo 205 cells unexpressed CXCR4 (*p*<0.001; Fig. 3C). The migration effect of CXCR7 was blocked by a CXCR7 antagonist CCX771 (*p*<0.001; Fig. 3C).

Taken together, these findings suggest that CXCR7 may significantly uphold colorectal carcinoma cell proliferation and promote cell migration.

### TLR4, MD-2, and CXCR7 are expressed in human colorectal carcinoma tissues

**Figure 4:**
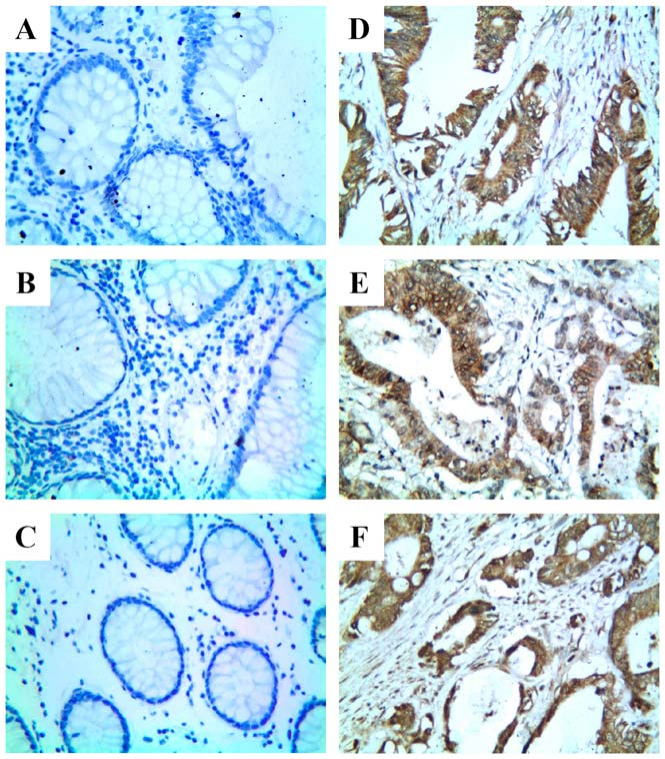
Representative examples of immunohistochemical staining of TLR4, MD-2, and CXCR7 in colorectal carcinoma tissues (original magnification 100×). Positive staining was observed as a dark brown color. Normal colorectal tissues showed negative immunohistochemical staining of TLR4 (A), MD-2 (B), and CXCR7 (C), and colorectal carcinoma tissues showed strong staining of TLR4 (D), MD-2 (E), and CXCR7 (F).

To further substantiate the growth-promoting and pro-migration effects of CXCR7 mediated by TLR4, we investigated the clinicopathologic significance of TLR4, MD-2 and CXCR7 expression using immunohistochemistry in human colorectal carcinoma tissues. TLR4, MD-2 and CXCR7 exhibited mostly cytoplasmic and plasmalemmal staining in colorectal carcinoma tissues (Fig. 4D, E, F). Normal tissue adjacent to tumor cells showed negative or occasionally weak staining that was mostly cytoplasmic (Fig. 4A, B, C). The differences in expression of the three molecules (TLR4, MD-2 and CXCR7) between normal colorectal tissues and colorectal carcinoma tissues were all found to be statistically significant (*p*<0.001, *p*<0.001, *p* = 0.034, resp.; Table 1).

**Table 1:** Correlation of TLR4, MD-2, and CXCR7 expression with clinicopathologic features in colorectal carcinoma.

Clinicopathologic	Case No.	TLR4 expression		*P* value	MD2 expression		*P* value	CXCR7 expression		*P* value
parameters		Low	High		Low	High		Low	High	
Total cases	66	31	35		20	46		47	19	
**Age**										
≤60	30	12	18	*p* = 0.3	8	22	*p* = 0.557	22	8	*p* = 0.728
>60	36	19	17		12	24		25	11	
**Tissue type**										
Normal colorectal tissue	20	20	0	*p*<0.001	15	5	*p*<0.001	19	1	*p* = 0.034
Colon carcinoma	66	31	35		20	46		47	19	
**Sex**										
Male	34	18	16	*p* = 0.316	9	25	*p* = 0.485	20	14	*p* = 0.022
Female	32	13	19		11	21		27	5	
**Tumor size**										
≤5 cm	26	18	8	*p* = 0.003	15	11	*p*<0.001	23	3	*p* = 0.013
>5 cm	40	13	27		5	35		24	16	
**TNM stage**										
I	2	2	0	*p* = 0.017	2	0	*p* = 0.013	2	0	*p* = 0.029
II	34	21	13		14	20		29	5	
III	18	5	13		2	16		9	9	
IV	12	3	9		2	10		7	5	
**Histologic grade**										
I	6	6	0	*p* = 0.002	5	1	*p* = 0.012	6	0	*p* = 0.004
II	50	24	26		13	37		38	12	
III	10	1	9		2	8		3	7	
**Lymph node metastasis**										
Negative	37	24	13	*p*<0.001	16	21	*p* = 0.010	33	4	*p*<0.001
Positive	29	7	22		4	25		14	15	
**Distant metastasis**										
Negative	51	29	22	*p* = 0.003	19	32	*p* = 0.027	38	13	*p* = 0.335
Positive	15	2	13		1	14		9	6	

### TLR4, MD-2 and CXCR7 expression is associated with clinicopathologic features

As shown in Table 1, there is a statistically significant correlation between TNM stage and TLR4 expression (*p* = 0.017), MD-2 expression (*p* = 0.013) or CXCR7 expression (*p* = 0.029). We also found that the increased expression is significantly associated with advanced histological grade (*p* = 0.002 for TLR4, *p* = 0.010 for MD-2 and *p* = 0.004 for CXCR7). There were no statistically significant differences in these molecules with regard to patient age and sex.

### Single or combined high expression of TLR4, MD-2 and CXCR7 are associated with tumor size and lymph node metastasis

The incidence of tumor size tended to be higher in cases with high rather than low expression of TLR4, MD-2, and CXCR7 (*p* = 0.003, *p*<0.001, *p* = 0.013, resp.; Table 1). Notably, when compared with high expression of these single markers, concomitant and high expression of any two of the three markers was statistically significantly associated with tumor size in patients with colorectal carcinoma (*p*<0.001 for TLR4/MD-2, *p* = 0.001 for TLR4/CXCR7 and *p*<0.001 for MD-2/CXCR7; Table 2). We also found that combined high expression of all TLR4, MD-2, and CXCR7 was significantly associated with tumor size, when compared with other combinations (*p* = 0.037; Table 2).

**Table 2:** Correlation of combined high expression of TLR4, MD-2, and CXCR7 with tumor size and metastasis.

	Tumor size		*p* -value	Lymph node metastasis		*p* value	Distant metastasis		*p* value
	≤5 cm(%)	>5 cm(%)		Negative(%)	Positive(%)		Negative(%)	Positive(%)	
**TLR4/MD2**									
(1)Both TLR4/MD2 low expression	13(81)	3(19)	*p*<0.001	14(87)	2(13)	*p*<0.001	15(94)	1(6)	*p* = 0.081
(2)One of TLR4/MD2 high expression	12(34)	23(66)	(2) versus (3)	21(62)	13(38)	(2) versus (3)	27(77)	8(23)	(2) versus (3)
(3)Both TLR4/MD2 high expression	1(7)	14(93)	*p* = 0.076	2(13)	13(87)	P = 0.002	9(60)	6(40)	*p* = 0.304
**TLR4/CXCR7**									
(1)Both TLR4/CXCR7 low expression	14(67)	7(33)	*p* = 0.001	19(90)	2(10)	*p*<0.001	21(100)	0(0)	*p* = 0.009
(2)One of TLR4/CXCR7 high expression	12(34)	23(66)	(2) versus (3)	18(53)	16(47)	(2) versus (3)	24(69)	11(31)	(2) versus (3)
(3)Both TLR4/CXCR7 high expression	0(0)	10(100)	*p* = 0.042	0(0)	10(100)	*p* = 0.003	6(60)	4(40)	*p* = 0.710
**MD2/CXCR7**									
(1)Both MD2/CXCR7 low expression	11(100)	0(0)	*p*<0.001	11(100)	0(0)	*p*<0.001	11(100)	0(0)	*p*<0.001
(2)One of MD2/CXCR7 high expression	11(38)	18(62)	(2) versus (3)	18(62)	11(38)	(2) versus (3)	26(90)	3(10)	(2) versus (3)
(3)Both MD2/CXCR7 high expression	4(15)	22(85)	*p* = 0.061	8(31)	18(69)	*p* = 0.020	14(54)	12(46)	*p* = 0.003
**TLR4/MD2/CXCR7**									
(1)Fewer than TLR4,MD2 and CXCR7 high expression	26(44)	33(56)	*p* = 0.037	37(64)	21(36)	*p* = 0.002	48(81)	11(19)	*p* = 0.042
(2)All of TLR4,MD2 and CXCR7 high expression	0(0)	7(100)		0(0)	7(100)		3(43)	4(57)	

At the same time, the incidence of lymph node metastasis tended to be higher in patients with colorectal carcinoma with high rather than low expression of TLR4, MD-2, or CXCR7 (*p* = 0.001, *p* = 0.012, *p*<0.001, resp.; Table 1). After adjusting for age, sex, tumor size and histologic grade, the risk of lymph node metastasis was significantly associated with CXCR7 (OR adjusted = 14.06; 95% CI, 1.09–180.50; Table 3). As shown in Table 2, there were statistically significant differences in high expression of these single molecules, and high expression of two of the three molecules TLR4/MD-2, TLR4/CXCR7, TLR4/CXCR7 with regard to lymph node metastasis in patients with colorectal carcinoma (*p*<0.001, *p*<0.001, *p* = 0.001, resp.). Combined high expression of all three molecules was significantly associated with lymph node metastasis in patients as compared with cases not showing such expression (*p* = 0.002; Table 2).

**Table 3:** The simple and multiple logistic regression model analyzing the predictors of colorectal carcinoma lymph node metastasis.

Variable	Simple logistic regression		Multiple logistic regression*	
	OR	95% CI	OR	95% CI
TLR4	5.80	2.00–17.18	8.77	1.18–65.08
MD2	4.76	1.38–16.45	2.65	0.23–31.04
CXCR7	8.84	2.49–31.41	14.06	1.09–180.50

*ORs and 95% CIs were calculated by unconditional logistic regression after adjusting for age, sex, tumor size and histologic grade.

In addition, the incidence of distant metastasis tended to be higher in patients with colorectal carcinoma with high rather than low expression of TLR4 or MD-2 (*p* = 0.003, *p* = 0.027, resp.; Table 1). Moreover, there are statistically significant differences in high expression of single TLR4, MD-2, or CXCR7 molecules, and high expression of both of TLR4/CXCR7, or MD-2/CXCR7 molecules with regard to distant metastasis in patients with colorectal carcinoma (*p* = 0.009, *p* = 0.001, resp.; Table 2). However, this result did not apply in the case of combination of TLR4/MD-2 (*p* = 0.081; Table 2). Combined high expression of all three molecules is significantly associated with distant metastasis in patients with colorectal carcinoma as compared with cases of high expression in fewer than all three (*p* = 0.042; Table 2).

## Discussion

In this study, we show that LPS-TLR4-MD-2 induced CXCR7 expression alteration in the colorectal carcinoma SW480 and Colo 205 cell lines. CXCR7 mediated by TLR4 modulated tumor cell proliferation and migration. However, exposure of SW480 and Colo 205 cell lines to LPS had no effect on CXCR4 expression. Furthermore, higher rates of TLR4, MD-2, and CXCR7 expression were found in colorectal carcinoma tissues than in normal tissues. There was a statistically significant correlation between single TLR4, MD-2, and CXCR7 expression levels and human colorectal carcinoma TNM stage, advanced histological grade, tumor size and lymph node metastasis. Furthermore, it was demonstrated that concomitant expression of the three molecules TLR4, MD-2 and CXCR7 is associated with increased carcinoma growth and metastasis potential in human colorectal carcinoma.

The link between inflammation and cancer suggests that the mechanisms contributing to inflammation may also be critical for tumor formation and progression. TLRs comprise a group of pattern recognition receptors which can sense pathogen invasion. TLR4 is expressed in a variety of tumors, and TLR4 activation can promote development and progression, apoptosis resistance, invasion, metastasis of tumors and tumor immune escape [16]–[17]. Among all the TLR4 accessory molecules, MD-2 is the only one that is absolutely required for the response to LPS [18]–[20]. Pathogens have devised many strategies to evade or manipulate TLR4–MD-2 activity. Once LPS binds to TLR4 two signaling pathways are activated: a MyD88-dependent pathway and a MyD88-independent pathway. Based on studies using macrophages, these pathways are responsible for expression of NF-kB/pro-inflammatory cytokines [21]–[23], can alter some cell functions: cytoprotection, apoptosis, etc. In our present work, knockdown of MD-2 in SW480 and Colo 205 cell lines has indeed provided evidence that MD-2 is indispensable for TLR4 signaling. Simultaneously, LPS had no effect on CXCR7 or CXCR4 expression of HT-29 cells not expressing MD2, with expression of only TLR4. Moreover, the differences in expression of TLR4 and MD-2 between normal colorectal tissues and colorectal carcinoma tissues were all found to be statistically significant. Increased expression of TLR4 and MD-2 was significantly associated with TNM stage and advanced histological grade. Concomitant and high expression of both markers TLR4/MD-2 was more predictive of tumor size and lymph node involvement.

The development of metastasis is complex, requiring multiple individual steps to successfully establish a tumor at a secondary site. The process requires a tumor cell to acquire the ability of proliferation, anti-apoptosis, migration and invasion, et al. Increasing evidence suggests that chemokines and their receptors are closely related to the mediation of inflammation, chemotaxis of white blood cells and tumor biology. CXCR4/CXCL12, induced by pro-inflammatory cytokines plays a role in metastasis of tumors by inducing chemotactic, proliferative and invasive response [24]. The second chemokine receptor of CXCL12, CXCR7 was formerly known as RDC1 [25]. As a membrane associated receptor protein, CXCR7 is expressed on the surface of many tumor cells [26]–[28], activated endothelial cells, and fetal liver cells, but rarely in other normal cells. There is growing discussion about CXCR7 enhancing tumor growth and migration through various signaling pathways [26]–[27]. However, some scientists consider that CXCR7 is a non-signaling receptor (decoy receptor) [28], which binds the ligands CXCL12 and ITAC/CXCL11, does not activate typical G*α*i pathways of a chemokine receptor that would result in GTP hydrolysis or calcium mobilization [27]. Therefore, we became interested in the role of the CXCR7/CXCL12 axis in the biologic processes of colorectal carcinoma. Moreover, we tried to verify the relationship between TLR4-MD-2 and CXCR7, which has not been previously investigated.

Our data showed a marked increase of CXCR7 expression in a TLR4/MD-2 positive colorectal carcinoma SW480 and Colo 205 cell lines in response to LPS. Knockdown of MD-2 inhibited LPS-mediated time- and dose-dependent CXCR7 expression alterations. Furthermore, exposure of SW480 and Colo 205 cell lines to LPS had no effect on CXCR4 expression. CXCR7 and CXCR4 expressed on tumor cells significantly induced proliferation and migration in the presence of CXCL12. However, in this study, the proliferative effect of CXCR7 is more than that of CXCR4, there was no significant difference in migration effect of CXCR7 and CXCR4. Then, the differences in expression of CXCR7 between normal colorectal tissues and colorectal carcinoma tissues were found to be statistically significant. There was a statistically significant correlation between CXCR7 expression and TNM stage and advanced histological grade. We also found CXCR7 was an independent predictor of metastasis - independent of size and histological grade.

Notably, the present study suggests that TLR4, MD-2, and CXCR7 are highly expressed in colorectal carcinoma. Furthermore, the concomitant and high expression of all three markers is significantly associated with tumor size, lymph node metastasis and distant metastasis. However, more evidence will be needed to identify the mechanism of TLR4-MD-2 activation through CXCR7-mediated signaling and their synergistic role in tumors.

In summary, we have shown that the alteration of CXCR7 expression mediated by TLR4 promotes tumor cell proliferation and migration in human colorectal carcinoma. This suggests that infective states are not merely related to surrounding inflammatory products but also to an effect of LPS on occurrence and development of colorectal carcinoma. Crosstalk among the three proteins TLR4, MD-2, or CXCR7 may be closely related to promotion of tumor growth and metastasis. Inhibition of TLR4, MD-2, or CXCR7 may be an effective adjuvant therapy for colorectal carcinoma.

## Materials and Methods

### Cell lines and culture

The following human colorectal carcinoma cell lines were from American type culture collection (ATCC): Caco-2, HT-29, DLD-1, Colo 205, HCT 116, RKO, SW480 and SW620.

Caco-2 cells were cultured in Eagle's Minimum Essential Medium (Catalog No. 30-2003, ATCC, USA) supplemented with 20% fetal bovine serum (FBS), penicillin (100 U/ml), and streptomycin (100 µg/ml) at 37°C and in 5% CO_2_. HT-29 cells and HCT 116 cells were cultured in McCoy's 5a Medium Modified (Catalog No. 30-2007, ATCC, USA) supplemented with 10% FBS, penicillin (100 U/ml), and streptomycin (100 µg/ml) at 37°C and in 5% CO_2_. DLD-1 cells and Colo 205 cells were cultured in RPMI-1640 Medium (Catalog No. 30-2001, ATCC, USA) supplemented with 10% FBS, penicillin (100 U/ml), and streptomycin (100 µg/ml) at 37°C and in 5% CO_2_. RKO cells were cultured in Eagle's Minimum Essential Medium (Catalog No. 30-2003, ATCC, USA) supplemented with 10% FBS, penicillin (100 U/ml), and streptomycin (100 µg/ml) at 37°C and in 5% CO_2_. SW480 cells and SW620 cells were cultured in Leibovitz's L-15 Medium (Catalog No. 41300039, Gibco, Invitrogen, Life Technologies, USA) supplemented with 10% FBS, penicillin (100 U/ml), and streptomycin (100 µg/ml) at 37°C and in 5% CO_2_.

### Patients and Specimens

This research was approved by Medical Ethics Committee of The First Affiliated Hospital of Bengbu Medical College. All participants gave informed written consent before participating in the study. Colorectal carcinoma samples were collected from 66 patients undergoing curative-intent surgery at the Department of Surgery, The First Affiliated Hospital of Bengbu Medical College, and the Department of Surgery, Renji Hospital, Shanghai Jiao Tong University School of Medicine between 2005 and 2011. There were also 20 normal colorectal tissue samples adjacent to colorectal carcinoma (used as controls). The histologic sections were reviewed by two expert pathologists to verify the histologic diagnosis. None of the patients had received any preoperative treatment. Tumors were staged according to the American Joint Committee on Cancer (AJCC) pathologic tumornode-metastasis (TNM) classification. Informed consent was obtained from all study subjects before sample collection and these samples were used according to ethical standards.

### Antibodies and other reagents

LPS (Catalog No. L6011) and Polymyxin B (PMB; Catalog No. P4932), the LPS inhibitor, were from Sigma-Aldrich, USA. Monoclonal anti-human TLR4 antibody (Clone ab22048), monoclonal anti-human MD-2 antibody (Clone ab24182) were from Abcam, England; monoclonal anti-human CXCR7 antibody (Clone MAB4227) from R&D systems, USA; monoclonal anti-human CXCR4 antibody (Clone 5555974) from BD, USA; a CXCR4 antagonist (AMD3100) from Sigma-Aldrich, USA; CXCL12 (Clone 2716-SD-025/CF) from R&D, USA. A CXCR7 antagonist (CCX771) was kindly provided by ChemoCentryx. HRP-conjugated secondary antiserum (Clone A0216, Beyotime, China) was used in Western blotting assay.

### Reverse transcriptase (RT)-PCR and real-time quantitative-PCR assay

Total RNA was isolated from colorectal carcinoma cell lines using TRIzol (Invitrogen, USA) according to the manufacturer's instructions, and reverse transcribed. The primers for TLR4 were 5′-TGCAATGGATCAAGGACCAGAGG-3′ and 5′-TGCAGCCAGCAAGAAGCATCAG-3′. The primers for MD-2 were 5′-CCGAGGATCTGATGACGATT A-3′ and 5′-GGCTCCCAGAAATAGCTTCAA -3′. The primers for CXCR7 were 5′- CACAGCACAGCCAGGAAGG-3′ and 5′-GTTCCCTGGCTCTGAGTAGTCGA-3′. PCR products were analysed by electrophoresis in 2% agarose gel stained with ethidium bromide. The real-time quantitative-PCR reaction was performed using SYBR Green master mix (ABI, Foster City, CA). GAPDH gene was used as an endogenous control for sample normalization. The primers for GAPDH were 5′-GGATTTGGTCGTATTGGG-3′ and 5′-GGAAGATGGTGATGGGATT-3′. Data were collected and quantitatively analyzed on an ABI Prism 7900 sequence detection system. Differences in the relative levels of three markers normalized to GAPDH can be estimated by differences in the ratios.

### Flow cytometric assay

For in vitro studies of CXCR7 and CXCR4 expression regulation, all cell lines were cultured in 2% FBS. After 12 h, colorectal carcinoma SW480, Colo 205 and HT-29 cells were treated with LPS in the presence or absence of PMB. Cultures were trypsinized and 5×10^5^ cells were incubated for 1 h with a monoclonal anti-human CXCR7 or CXCR4 antibody, and analysed using a Becton Dickinson FACSCan with CellQuest software. Cells from the SW480 and Colo 205 cell lines were isolated and gated to exclude dead and GFP-negative cells so that only cells that were GFP positive were analysed.

### Transient transfection

A sequence of 19-nucleotide residues in length (GUGGGAGAGAUUUAAAGCA) specific to the human MD-2 cDNA (nucleotide residues, 222–241) was selected for synthesis of a siRNA (GenenPharma, USA). The siRNA was dissolved in DEPC water and transfected into colorectal carcinoma SW480 and Colo 205 cell lines with Lipofectamine 2000 (Invitrigen, USA). Effects of siRNA for MD-2 was compared with those of a random siRNA sequence (negative control sequence). The depletion of endogenous MD-2 by the siRNA was confirmed by RT-PCR and Western blot.

### Western blot analysis

Colorectal carcinoma SW480 and Colo 205 cell lines were lysed in RIPA Lysis Buffer (Beyotime, China) supplemented with protease inhibitor Cocktail (Catalog No. A7706_0001, AppliChem, Germany). Protein concentration in the postnuclear lysates was measured by BCA Protein Assay (Beyotime, China) and equal amounts of protein lysates (60 µg) were loaded on 10% SDS-PAGE. Gels were transferred to nitrocellulose using iBlot Dry Blotting System (Invitrogen, USA). Filters were blocked with 5% dry skimmed milk and blotted with the specific primary antibodies: mouse monoclonal antibody to MD-2. Blots were then incubated with the appropriate HRP-conjugated secondary antiserum, and signal revealed with the WestPico chemiluminescence system (Pierce). Filters were stripped for 10 min with ReBlot Plus Strong Antibody Stripping Solution (Millipore).

### Cell proliferation assay

Colorectal carcinoma SW480 and Colo 205 cell lines were cultured in 96-well plates at an initial density of 2,000 cells per well, in 100 µl of 1% FBS-medium without (control) or with addition of CXCL12 (100 ng/ml). In some of the experiments, SW480 cells were pretreated (1 h) with CCX771 (1 UM) and/or AMD3100 (10 µg/ml), Colo 205 cells were pretreated (1 h) with CCX771 (1 UM). After 24 h, and 48 h, effects of CXCL12 on cell proliferation were determined by using a WST-1 Kit (Beyotime, China). Each experimental condition was sampled in duplicate and the experiments were repeated five times.

### Apoptosis assay

Colorectal carcinoma SW480 and Colo 205 cell lines were incubated in 1% FBS-medium without (control) or with addition of CXCL12 (100 ng/ml). In some of the experiments, SW480 cells were pretreated (1 h) with CCX771 (1 UM) or/and AMD3100 (10 µg/ml), Colo 205 cells were pretreated (1 h) with CCX771 (1 UM). After 24 h and 48 h, the cells were washed with incubation buffer, and incubated for 30 min at room temperature with 0.5 mg/ml propidium iodide (eBioscience, USA) and annexin V-FITC (eBioscience, USA). 2×10^5^ cells were collected for each sample by flow cytometry. Each experiment was repeated five times.

### Migration experiments

Colorectal carcinoma SW480 and Colo 205 cell lines were resuspended in 1% FBS-medium of 5×10^5^ cells/ml, and seeded into the upper chambers of Transwell inserts (Millipore). 1% FBS-medium was added to the lower chambers, without (control) or with addition of CXCL12 (100 ng/ml). In some of the experiments, SW480 cells were pretreated (1 h) with CCX771 (1 UM) or/and AMD3100 (10 µg/ml), Colo 205 cells were pretreated (1 h) with CCX771 (1 UM).. The migration was carried out at 37°C and 5% CO_2_ for 24 h. After incubation, the nonmigrated cells were removed from the upper surface of the filters, and the migrated cells, adherent to the lower surface, were counted (ten high-power fields/well). Each experiment was repeated five times.

### Immunohistochemistry assay

Sections were subjected to routine deparaffination and rehydration. Antigen retrieval was achieved by microwaving in 0.01 mol/L citrate buffer for 10 min and then cooling for 30 min. The endogenous peroxidase activity was inhibited by incubation with 3% hydrogen peroxide in methanol for 20 min and nonspecific binding was blocked by incubation with 5% bovine serum albumin in phosphate-buffered saline (PBS) at room temperature. After three PBS washes, the specimens were reacted overnight at 4°C with murine anti-human monoclonal antibodies: anti-TLR4, anti-MD-2 and anti-CXCR7. After incubation with rat anti-mouse-IgG2b-horseradish peroxidase, signal was developed with 3,30-diaminobenzidine tetrahydrochloride in Tris–HCl buffer (pH 7.6) containing 0.02% hydrogen peroxide. The sections were then counterstained with hematoxylin and mounted. Negative controls were performed by replacing the primary antibody with nonspecific IgG at the same concentration.

### Interpretation and evaluation of immunohistochemical results

Immunostaining was independently examined by two clinical pathologists who were unaware of the patient outcome. For each sample, five high-power fields (100×) were randomly selected. Staining intensity and percentage of positive tumor cells were assessed. The extent of the staining was categorized into five semiquantitative classes based on the percentages of positive tumor cells: 0 (<5% positive cells), 1 (6–25% positive cells), 2 (26–50% positive cells), 3 (51–75% positive cells) and 4 (>75% positive cells). The intensity of cytoplasmic and membrane staining was also determined semiquantitatively on a scale of 0–3 as follows: 0 (negative), 1 (weakly positive), 2 (moderately positive) and 3 (strongly positive). A consensus score was assigned for each section after discussion and careful review of all slides by the two pathologists. Multiplication of the intensity and the percentage scores gave rise to the final staining score: 0 (negative), + (1–4), ++ (5–8), and +++ (9–12). For statistical analysis, tumors having a final staining score of negative or +, which showed a weak or moderate/strong immunoreactivity were grouped into a low expression group and were compared to tumors with scores of ++ or +++ as the high expression group.

### Statistical analysis

Differences were evaluated using Statistical Package for Social Science software (version 16.0, SPSS Inc., Chicago, IL). The association of staining intensity with clinicopathologic patterns was assessed with the Chi square test and two-sided Fisher's exact test to determine the significance of the difference between the the covariates. A logistic regression model was used to estimate odds ratios (ORs) and 95%confidence intervals (CIs) for the association between CXCR7 and ymph node metastases. All measurement data are presented as mean ± SEM. Statistical significance was evaluated by one-way ANOVA, followed by least significant difference (LSD) test. P values<0.05 were considered to be statistically significant.
